# The complete chloroplast genome sequence of *Quercus sessilifolia* Blume (Fagaceae)

**DOI:** 10.1080/23802359.2021.2017366

**Published:** 2022-01-10

**Authors:** Shuifei Chen, Wenwen Zhang, Yao Li, Xiaomin Ge, Xu Zhou, Yaping Hu, Hui Ding

**Affiliations:** a Research Center for Biodiversity Conservation and Biosafety/State Environmental Protection Scientific Observation and Research Station for Ecological Environment of Wuyi Mountains/Biodiversity Comprehensive Observation Station for Wuyi Mountains/State Environmental Protection Key Laboratory on Biosafety, Nanjing Institute of Environmental Sciences, Ministry of Ecology and Environment of China, Nanjing, China; b Co-Innovation Center for Sustainable Forestry in Southern China, College of Biology and the Environment, Key Laboratory of State Forestry and Grassland Administration on Subtropical Forest Biodiversity Conservation, Nanjing Forestry University, Nanjing, China

**Keywords:** Chloroplast genome, Fagaceae, phylogeny; *Quercus sessilifolia*

## Abstract

*Quercus sessilifolia* Blume is one of the dominant tree species in East Asian evergreen broadleaved forests. In this study, we assembled and characterized the plastome of *Q. sessilifolia* using Illumina paired-end data. The circular genome is 160,813 bp in size, consisting of two copies of inverted repeat (IR) regions of 25,862 bp, one large single-copy (LSC) region of 90,218 bp, and one small single-copy (SSC) region of 18,871 bp. It encodes a total of 113 unique genes, including 79 protein-coding genes, 30 tRNA genes, and four rRNA genes. Phylogenetic analysis based on 28 chloroplast genome sequences indicated that *Q. sessilifolia* was most closely related to *Q. myrsinifolia* with 90% bootstrap support.

*Quercus sessilifolia* Blume, one of the dominant tree species in East Asian evergreen broadleaved forests, has a widespread distribution across Japan, Taiwan, and mainland China. It is phylogenetically closely related to *Q. acuta*, *Q. ciliaris*, *Q. arbutifolia*, *Q. stewardiana*, and *Q. kiukiangensis*, which comprise the Acuta clade in the single-celled trichome base (STB) lineage of *Quercus* section *Cyclobalanopsis* (ring-cupped oaks, Deng et al. 2018). Previous studies have shown that *Q. sessilifolia* and its Japanese sibling, *Q. acuta*, can produce genetically admixed individuals through introgressive hybridization (Tamaki and Okada 2014). In subtropical China, *Q. sessilifolia* can coexist with *Q. ciliaris* and *Q. stewardiana*, but it remains unclear whether interspecific gene flow occurs among them. Here, we sequenced, assembled, and annotated the complete chloroplast (cp) genome of *Q. sessilifolia* to provide more useful genomic resources for the future population genetic studies of closely related ring-cupped oaks in the Acuta clade.

Fresh young leaves of *Q. sessilifolia* were sampled from an adult tree growing at Nanjing Forestry University, Nanjing, Jiangsu, China (32.08°N, 118.81°E). The plant material was collected with permission of Nanjing Forestry University. The voucher specimen was preserved at the Herbarium of Nanjing Forestry University (HNFU, https://www.cvh.ac.cn/ins/info.php?code=NF, Xiangui Yi, 354067272@qq.com) under the accession number 21012601. Total genomic DNA was extracted from silica-dried leaves using the CTAB method (Doyle and Doyle, 1990). Whole genome sequencing was conducted with the Illumina NovaSeq 6000 platform by Nanjing Genepioneer Biotechnologies Inc. (Nanjing, China). A total of 27,882,958 clean reads were produced and used for the *de novo* assembly with SPAdes 3.10.1 (−k 55, 87, 121; Bankevich et al. 2012), SSPACE 2.0 (Boetzer et al. 2011), and GapFiller 2.1.1 (Nadalin et al. 2012). Gene annotation was performed using Prodigal 2.6.3 (Hyatt et al. 2010), Hmmer 3.1b2 (Johnson et al. 2010), and Aragorn 1.2.38 (Laslett and Canback 2004).

The complete cp genome of *Q. sessilifolia* (GenBank accession number MZ382817) is a circular molecule of 160,813 bp in length, consisting of one large single-copy (LSC) region (90,218 bp), one small single-copy (SSC) region (18,871 bp), and two copies of inverted repeat (IR) regions (25,862 bp). The overall GC content was 36.90%, while the corresponding values of the LSC, SSC, and IR regions were 34.75%, 31.13%, and 42.75%, respectively. The cp genome encoded a total of 131 genes, of which 113 were unique and 18 were duplicated in the IR regions. The 113 unique genes contained 79 protein-coding genes, 30 tRNA genes, and four rRNA genes.

To identify the phylogenetic position of *Q. sessilifolia*, a maximum-likelihood (ML) tree of 26 oak species and two outgroups, *Trigonobalanus doichangensis* (A.Camus) Forman and *Fagus crenata* Blume, was inferred under the GTRGAMMA substitution model using raxmlGUI 1.5 (Silvestro and Michalak 2012). The GenBank accession numbers for the used complete cp genome sequences are shown in Figure 1. The HomBlocks pipeline (-min = 200 -method = Gblocks; Bi et al. 2018) was utilized to recognize locally collinear blocks among cp genomes and excavate phylogeny informative regions. Five locally collinear blocks were identified and a matrix of 99,249 bp was generated. Node support was assessed by using 1,000 fast bootstrap replicates. Our results indicated that *Q. sessilifolia* was among the members of *Quercus* section *Cyclobalanopsis*, and was most closely related to *Q. myrsinifolia* with 90% bootstrap support (Figure 1).

**Figure 1.**
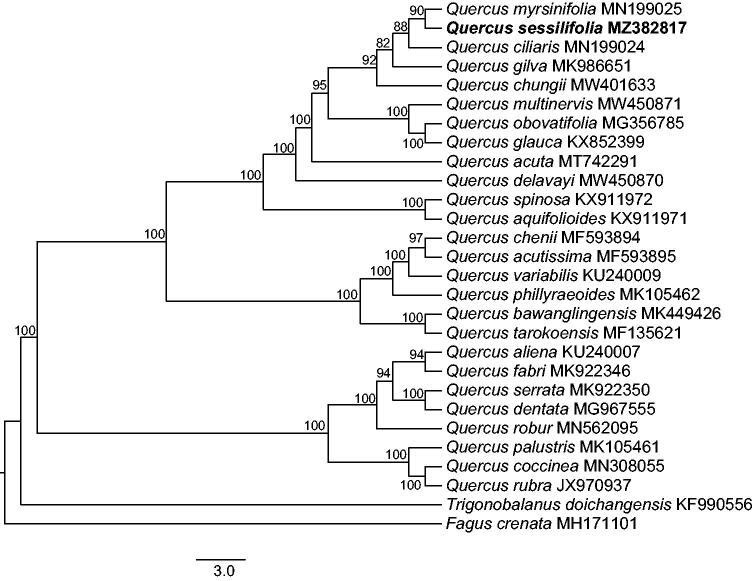
The maximum-likelihood (ML) phylogenetic tree reconstructed by raxmlGUI 1.5 (Silvestro and Michalak 2012) based on cp genome sequences of 26 oak tree species and two outgroups, *Trigonobalanus doichangensis* (A.Camus) Forman and *Fagus crenata* Blume. The bootstrap support value is labeled for each node.

## Data Availability

The genome sequence data that support the findings of this study are openly available in GenBank of NCBI at https://www.ncbi.nlm.nih.gov/ under the accession no. MZ382817. The associated BioProject, SRA, and Bio-Sample numbers are PRJNA739391, SRR14866431, and SAMN19782876, respectively.
